# The Meckel-Gruber Syndrome protein TMEM67 (meckelin) regulates basal body planar polarization and non-canonical Wnt signalling via Wnt5a and ROR2

**DOI:** 10.1186/2046-2530-4-S1-P40

**Published:** 2015-07-13

**Authors:** Z Abdelhamed, S Natarajan, C Inglehearn, C Toomes, C Johnson, D Jagger

**Affiliations:** 1 Anatomy and Embryology Department, Al-Azhar University, Cairo, Egypt; 2 Ophthalmology and Neuroscience, University of Leeds, Leeds, UK; 3 Ear Institute, University College London, London, UK

## Objective

Ciliopathies are a group of developmental disorders that manifest with multi-organ anomalies. Mutations in *TMEM67 *have been reported in human ciliopathies that include Meckel-Gruber and Joubert syndromes. In this study we describe multi-organ developmental abnormalities in the *Tmem67^tm1Dgen/H1 ^*knockout mouse that closely resemble those of *Wnt5a *and *Ror2 *knockout mice.

## Methods

We used anatomical assessment, immunofluorescence confocal microscopy and biochemical methods to determine mutant phenotypes at the organismal, cellular and molecular levels.

## Results

*Tmem67^-/- ^*mutant phenotypes include pulmonary hypoplasia, ventricular septal defects, shortening of the body longitudinal axis, limb abnormalities, and cochlear hair cell stereociliary bundle orientation and basal body/kinocilium positioning defects. The basal body/kinocilium complex was often uncoupled from the hair bundle, suggesting aberrant basal body migration. TMEM67 (meckelin) is essential for phosphorylation of the non-canonical Wnt receptor ROR2 (receptor tyrosine kinase-like orphan receptor 2) upon Wnt5a stimulation. ROR2 interacts with the intracellular C-terminal domain of TMEM67 and co-localizes with TMEM67 at the ciliary transition zone. The N-terminal domain of TMEM67 preferentially binds to Wnt5a in an *in vitro *binding assay. *Tmem67 *mutant embryonic lungs in *ex vivo *culture failed to respond to Wnt5a stimulation of epithelial morphogenesis. However, stimulating the non-canonical Wnt pathway downstream of the receptor by activating RhoA resulted in an elicited response and the rescue of lung hypoplasia phenotypes.

## Conclusion

Our data suggest that TMEM67 is a novel receptor that has a major role in non-canonical Wnt signalling by Wnt5a and ROR2. We propose that this signalling ensures correct basal body positioning.

